# Genome-Wide Association Study of Rib Number and Total Thoracolumbar Vertebrae Number in a Jishen Black Pig Population

**DOI:** 10.3390/vetsci13080776

**Published:** 2026-08-03

**Authors:** Yu He, Fengyi Dong, Long Jin, Jiayi Ning, Wuyang Liu, Chengyue Feng, Zhikai Zhu, Han Sun, Xiaoran Zhang, Changyi Chen, Luyao Bie, Boxing Sun, Hao Sun, Chunyan Bai

**Affiliations:** College of Animal Science, Jilin University, Changchun 130062, China; heyu21@mails.jlu.edu.cn (Y.H.); dongfy25@mails.jlu.edu.cn (F.D.); jinlong25@mails.jlu.edu.cn (L.J.); ningjy24@mails.jlu.edu.cn (J.N.); wuyang24@mails.jlu.edu.cn (W.L.); cyfeng25@mails.jlu.edu.cn (C.F.); zhuzk9922@mails.jlu.edu.cn (Z.Z.); sunhan9923@mails.jlu.edu.cn (H.S.); xiaoran24@mails.jlu.edu.cn (X.Z.); cychen24@mails.jlu.edu.cn (C.C.); bieluyao@jlu.edu.cn (L.B.); sunpathing@vip.163.com (B.S.)

**Keywords:** Jishen Black pig, rib number, total number of thoracolumbar vertebrae, genome-wide association study, *VRTN*, heritability

## Abstract

The Jishen Black pig is a synthetic breed incorporating the genetic backgrounds of Chinese indigenous pigs and Western lean-type pigs. The objective of this study was to characterize phenotypic variation, estimate genetic parameters, and identify genomic loci associated with rib number (NR) and the total number of thoracolumbar vertebrae (NTLV) in Jishen Black pigs. NR, NTLV, and lumbar vertebra number (NLV) were measured in 389 pigs, and genotyping was performed using a 70K SNP chip. Genetic parameters were estimated using HIBLUP, and genome-wide association studies (GWAS) were conducted using the MLM and BLINK models in GAPIT v3.0. The mean values of NR, NTLV, and NLV were 14.88, 20.94, and 6.05, respectively, and their heritability estimates were 0.568, 0.493, and 0.140, respectively. GWAS identified a shared major association peak for NR and NTLV on SSC7 at 91.19–98.15 Mb, centered on *VRTN* and the adjacent *ABCD4-LTBP2-AREL1-PGF* linkage region, among which the loci at 96.12 Mb, 96.56 Mb and 97.79 Mb are NR-specific loci. Additional signals included *MIB1* on SSC6 and *MMRN2* on SSC14 for NTLV, together with NR-specific loci on SSC7 at 111.59 Mb and on SSC8. Database annotation indicated that several significant SNPs overlapped with records for rib number, carcass length, loin muscle area, backfat thickness, or teat number. This study identifies major genomic regions associated with vertebral-number traits in Jishen Black pigs and expands current knowledge of their genetic basis.

## Simple Summary

Rib number and thoracolumbar vertebra number are important production traits in pigs because they affect body length, carcass length, and the yield of high-value meat cuts. In this study, 389 Jishen Black pigs were evaluated to determine the extent of variation in rib number and thoracolumbar vertebra number and to identify genomic regions that may influence these traits. The results showed that rib number and thoracolumbar vertebra number exhibited moderate to high heritability, whereas lumbar vertebra number showed limited variation and low heritability. Highly significant signals were identified mainly near the *VRTN* gene on chromosome 7, and several other significant loci on additional chromosomes were also detected. This study enriches our understanding of the genetic basis underlying variation in rib number and thoracolumbar vertebra number.

## 1. Introduction

The porcine vertebral column consists of cervical, thoracic, lumbar, sacral, and caudal vertebrae [1]; among these, the thoracolumbar vertebrae constitute the central structure linking the trunk and hindquarters, and their number directly determines body length and linear carcass dimensions [2]. The number of thoracic vertebrae (NTV) is identical to the number of ribs (NR), because each pair of ribs corresponds to one thoracic vertebra; therefore, NR and NTV are highly concordant at both the genetic and developmental levels. The total number of thoracolumbar vertebrae (NTLV), defined as the sum of thoracic and lumbar vertebrae, is an integrated trait with substantial economic value in pig breeding and production. From the perspective of slaughter and carcass processing, an increase in vertebral number is usually accompanied by an extension of body length and an increase in rib number; each additional vertebra can increase body length by as much as 8 cm [3]. This means that each carcass can yield more high-value cuts, such as ribs and loin muscle, thereby improving lean-meat percentage and overall economic return. Consequently, NTLV and NR are regarded as promising target traits in pig breeding, and their genetic improvement is therefore of considerable importance for developing commercial pig populations with higher meat yield.

NR and NTLV show pronounced breed and population differences in pigs. A recent study of a Landrace x Yorkshire crossbred population showed that NR and NTLV ranged from 14 to 16 ribs and from 19 to 23 vertebrae, respectively [4]. Pig breeds originating from different latitudes exhibit adaptive differentiation in genomic regions associated with body length, indicating that trunk-length-related traits are jointly influenced by long-term selection and genetic background [5]. Accordingly, the effects of vertebral number and its associated loci are often population-specific among indigenous breeds, synthetic breeds, and foreign lean-type breeds. Conducting GWAS in specialized synthetic pig populations can help supplement existing genetic data from conventional commercial pig populations.

Vertebral number and rib number generally exhibit high heritability, with previous studies reporting estimates as high as 0.60–0.62 [6] and the genetic correlation between NR and NTLV reaching 0.79–0.97 [7]. High heritability indicates that phenotypic variation in these traits is primarily governed by additive genetic effects, whereas environmental effects are relatively limited; therefore, rapid genetic progress may be achieved through either conventional breeding or genomic selection. This property makes NR and NTLV valuable target traits for molecular breeding in pigs. Early genotyping-based selection of individuals carrying favorable genotypes may improve the genetic level of a population within a relatively short period, thereby facilitating the development of commercial pigs with longer carcasses and higher lean-meat yield.

Genome-wide association study (GWAS) is a classical approach for dissecting the genetic architecture of complex traits; by scanning SNP markers across the whole genome, it systematically identifies chromosomal regions and candidate genes significantly associated with target phenotypes. At present, GWAS has been widely used to elucidate the genetic basis of rib number (NR) and total number of thoracolumbar vertebrae (NTLV) in pigs. QTL records deposited in the Pig Quantitative Trait Locus database (PigQTLdb) consistently demonstrate that the *VRTN* gene on chromosome 7 (SSC7) is the core genetic determinant regulating rib-number variation, and subsequent independent studies have further consolidated its status as a major-effect gene [8,9,10]. Mechanistic work has shown that *NR6A1* regulates Hox expression dynamics and vertebrate trunk development [11], while porcine association studies have implicated *NR6A1* polymorphisms in vertebral-number variation [12]. In addition, *ABCD4* on SSC7 and *GREB1L* and *MIB1* on SSC6 have been identified as candidate genes for rib number [13]. Genes such as *LTBP2*, *TGFβ3*, and *BMPR1A* have also been reported to be associated with vertebral-number variation in pigs [7,12], suggesting that the genetic regulation of NR and NTLV involves a complex polygenic network. The stability of these loci across different genetic backgrounds and their interactions with *VRTN* require further validation.

Jishen Black pig is a synthetic breed developed from Beijing Black pigs and Large White pigs as paternal and maternal lines, respectively, and was officially approved by the National Commission of Animal Genetic Resources in November 2017 [14]. Its paternal line, the Beijing Black pig, is a synthetic black pig breed with a long breeding history [15], whereas the maternal line, the Large White pig, shows superior performance in growth rate, lean-meat percentage, and reproductive traits [16]; accordingly, Jishen Black pigs integrate genetic information from Chinese indigenous breeds and Western lean-type breeds and therefore harbor abundant genetic variation. In the Jishen Black pig population, we observed clear phenotypic variation in rib number. To date, no study has systematically investigated the molecular genetic basis of rib number and thoracolumbar vertebral number variation in Jishen Black pigs. Against this background, the present study used 389 Jishen Black pigs as the research population and performed genome-wide scans to dissect the genetic architecture underlying variation in NTLV and NR, identify significant SNPs affecting these traits, and screen candidate genes and functional regions through database annotation. Considering that heterogeneity in genetic background among populations may lead to differences in QTL effects or even the emergence of novel loci, GWAS analysis in this synthetic population may uncover population-specific genetic signals. The results can not only provide theoretical and data support for genetic evaluation and marker-assisted selection of superior alleles in Jishen Black pigs, but also offer a valuable reference for exploring the molecular regulatory mechanisms of vertebral development in other pig populations.

## 2. Materials and Methods

### 2.1. Phenotypic Data Collection

A total of 389 healthy pigs of similar age were included in this study. Complete phenotypic records for NTLV, NLV, and NR were available for all animals. Before analysis, the phenotype records were checked for missing values and biologically implausible counts, and no records were excluded. All pigs were slaughtered according to industry standards, and trained technicians recorded NTLV, NLV, and NR by manual palpation after slaughter. NR was recorded on the left side of each carcass using a consistent single-side scoring convention.

### 2.2. Genotypic Data Collection

After phenotypic data collection, approximately 2 g of fresh longissimus dorsi muscle tissue was immediately collected from each pig, placed in centrifuge tubes containing 75% ethanol, and stored at −20 °C for genomic DNA extraction. Genomic DNA was extracted using a commercial kit (DC112, Vazyme Biotech, Nanjing, China); after extraction, DNA purity was assessed by spectrophotometry, using A260/A280 ratios of 1.8–2.0 and A260/A230 ratios of 2.0–2.2 as quality-control criteria. High-quality DNA samples from all 389 pigs were genotyped using a 70K SNP chip (Glbizzia Biosciences Co., Ltd., Jiaxing, China). The SNP chip initially contained 73,715 markers. Quality control was performed sequentially: restriction to autosomes 1–18 retained 70,868 SNPs (2847 excluded); filtering SNPs with a genotype-missing rate greater than 0.10 retained 61,859 SNPs (9009 excluded); applying MAF > 0.05 retained 52,882 SNPs (8977 excluded); and requiring Hardy–Weinberg equilibrium *p* ≥ 1 × 10^−6^ retained 52,482 SNPs (400 excluded). Individual-level genotype missingness was evaluated using the missing command in PLINK v1.90, and all 389 pigs had genotype missing rates below 0.10; therefore, no individuals were excluded. Missing genotypes were imputed using BEAGLE v5.5, and the final dataset of 52,482 autosomal SNPs was used for downstream analyses.

### 2.3. Heritability Estimation

Genetic parameters for NTLV, NR, and NLV were estimated using a mixed linear model implemented in HIBLUP v1.6.0 [17]. Because all of the animals formed a single contemporary management group, were raised on the same farm and in the same batch, were of similar age, and were slaughtered within a concentrated period, these factors did not form distinguishable fixed-effect classes in the available dataset. Therefore, no additional fixed effects for farm, batch, slaughter date, or age were included in the model. The analytical model was as follows:

*y* = *u* + *a* + *e*

In this model, *y* denotes the vector of phenotypic observations for NTLV, NR, or NLV; *u* is the overall mean; a is the vector of additive genetic effects, assumed to follow a multivariate normal distribution *a*~N(0, Gσ_a_^2^), where G is the genomic relationship matrix capturing genetic relatedness among individuals based on SNP data and σ_a_^2^ is the additive genetic variance; and e is the residual vector, assumed to follow *e*~N(0, Iσ_e_^2^), where I is the identity matrix and σ_e_^2^ is the residual variance. Heritability (*h*^2^) was calculated as follows:

*h*^2^ = σ_a_^2^/(σ_a_^2^ + σ_e_^2^) (1)

### 2.4. Genome-Wide Association Analysis

Genome-wide association analyses (GWASs) for NTLV and NR were performed using the MLM and BLINK models implemented in GAPIT v3.0 [18]. The two models were selected to provide complementary control and detection properties: MLM explicitly models the genomic relationship matrix to reduce confounding by relatedness, whereas BLINK uses an iterative multi-locus procedure that reduces dependence on a single-marker testing framework and is well suited to traits containing strong major-effect signals. For both MLM and BLINK, the first three principal components were included as covariates to control population structure. The PCA plot is shown in Figure S1. The significance threshold was defined by Bonferroni correction as *p* = 0.05/N (9.53 × 10^−7^), where N was the number of SNPs retained after quality control (52,482). Genomic inflation factors (λGC) were calculated from the genome-wide association test statistics for each trait-model combination and reported with the Q-Q plots.

### 2.5. Functional Annotation of Significant Loci

Significantly associated SNPs were mapped according to the *Sus scrofa* 11.1 reference genome (Ensembl, http://ensembl.org; accessed on 1 July 2026). Animal QTLdb (Release 59) [19] and the PigBiobank database [20] were used to annotate significant SNPs, with emphasis on whether they had been previously associated with rib number, vertebral number, carcass length, or related traits. In addition, Ensembl was used to map genes harboring or located near significant SNPs, and NCBI and PigBiobank/PigGTEx resources were consulted to investigate the functions of the relevant genes.

## 3. Results

### 3.1. Descriptive Statistics and Genetic Parameter Estimates for the Phenotypes

**Figure 1:**
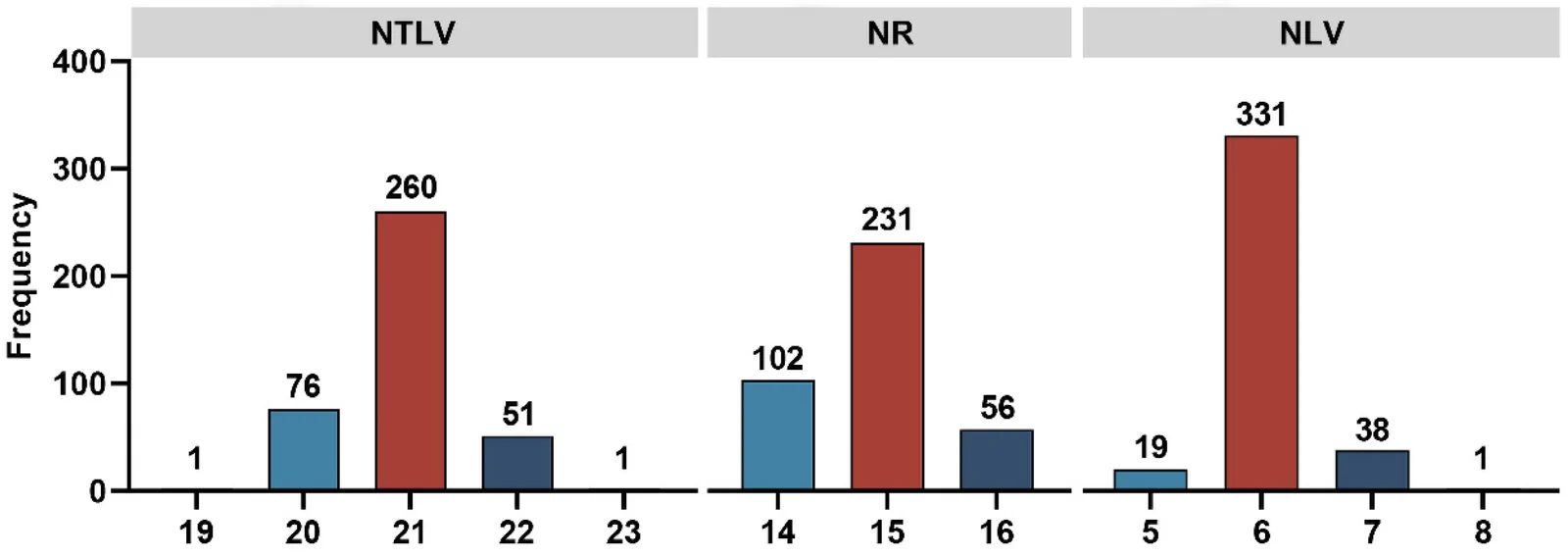
Population distributions of NTLV, NR, and NLV. Colors are used to distinguish the observed vertebral-number categories, with red highlighting the modal category for each trait.

Table 1 summarizes the descriptive statistics for NTLV, NR, and NLV in the 389 Jishen Black pigs, and Figure 1 shows the population distributions of the three traits. The mean values of NTLV, NR, and NLV were 20.94, 14.88, and 6.05, respectively, with phenotypic ranges of 19–23, 14–16, and 5–8, respectively. Both NR and NTLV showed clear discrete variation within the population. Most pigs had 15 ribs (*n* = 231), whereas 102 and 56 pigs had 14 and 16 ribs, respectively. Similarly, NTLV was mainly distributed at 21 vertebrae (*n* = 260), followed by 20 vertebrae (*n* = 76) and 22 vertebrae (*n* = 51), with only a few individuals having 19 or 23 vertebrae. By contrast, NLV was more concentrated, with 85.1% of individuals having six lumbar vertebrae (*n* = 331), indicating that variation in lumbar vertebral number was limited in this population and that differences in NTLV were mainly attributable to variation in thoracic vertebrae/rib number.

After quality control and imputation, a total of 52,482 autosomal SNPs were obtained from the genotyping data. Genetic-parameter estimation using the mixed linear model in HIBLUP v1.6.0 showed that NR and NTLV were traits with moderate to high heritability, with estimates of 0.568 and 0.493, respectively, suggesting that both traits are primarily regulated by additive genetic effects. The heritability of NLV was only 0.140, and its additive variance was low (0.022), indicating limited genetic variation in lumbar vertebral number in this population. Taken together, the phenotypic distribution and variance results indicate that, in the current Jishen Black pig population, breeding improvement of NTLV should prioritize major-effect loci related to rib number/thoracic vertebral number rather than loci related to lumbar vertebral number.

### 3.2. GWAS Results

GWASs for rib number (NR) and total number of thoracolumbar vertebrae (NTLV) were conducted using both MLM and BLINK models. The genome-wide significance threshold after Bonferroni correction was *p* = 9.53 × 10^−7^ (0.05/52,482). The λGC values were 0.924 for NR-MLM, 1.025 for NR-BLINK, 0.989 for NTLV-MLM, and 1.168 for NTLV-BLINK; the first three values were close to 1, whereas the NTLV-BLINK result showed modest inflation and was therefore interpreted together with the MLM, LD, and functional-annotation evidence. Overall, significant SNPs were located on SSC6, SSC7, SSC8, and SSC14, with SSC7:91.19–98.15 Mb representing the shared major-effect association region. The major SSC7 signal was concordant across models: *rs709317845* was detected by both models for NR, and *rs1113113109* was detected by both models for NTLV. The BLINK-specific signals *rs327309750* on SSC6 and *rs3471622288* on SSC14 were relatively independent signals for NTLV, whereas *rs319413074* on SSC7 at 111.59 Mb and *rs345314475* on SSC8 were detected only for NR and lie outside the shared SSC7:91.19–98.15 Mb interval. Database annotation indicated that several significant SNPs overlapped records for rib number, carcass length, loin muscle area, backfat thickness, or teat number.

**Figure 2:**
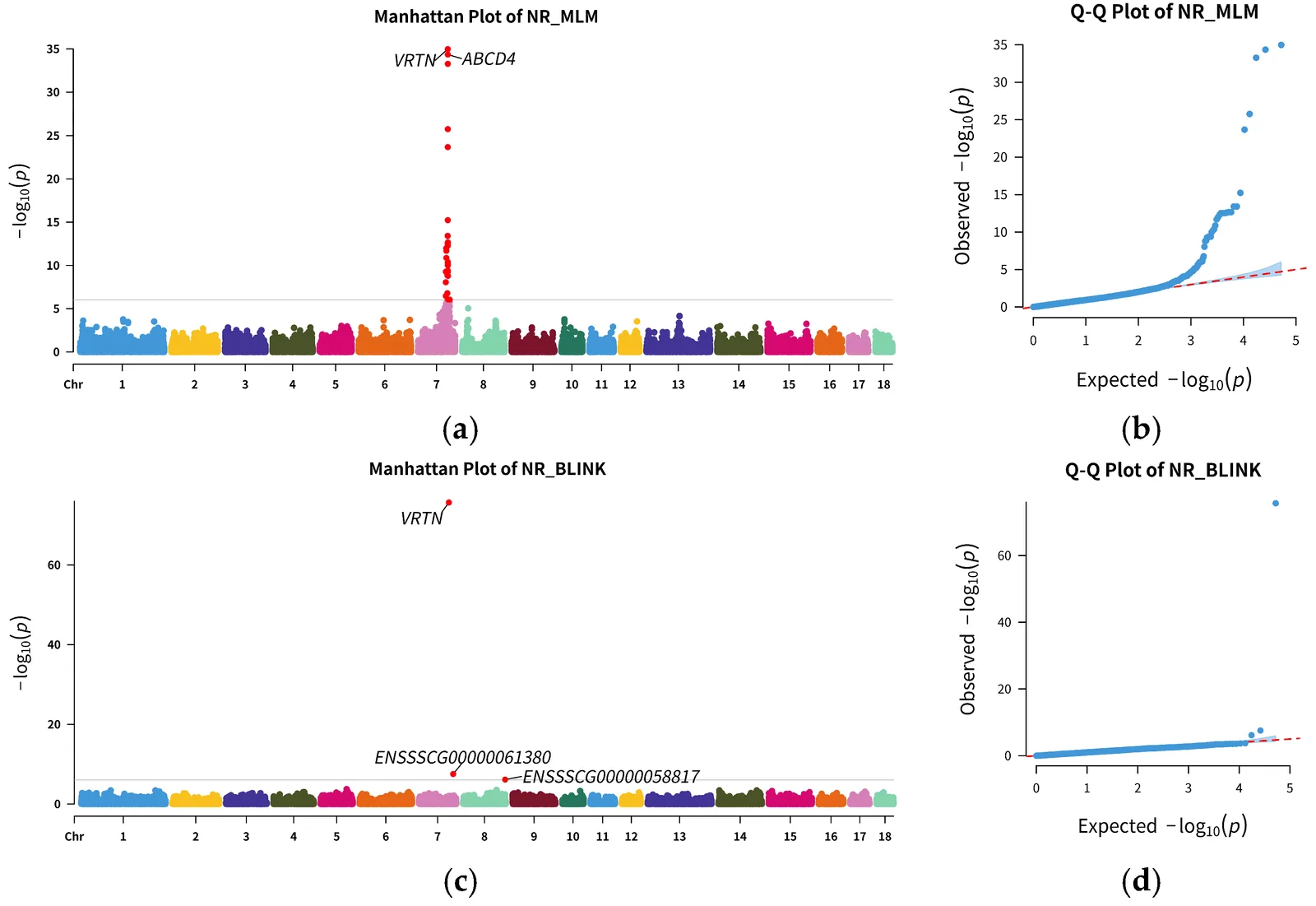
Manhattan and Q-Q plots for rib number (NR). (**a**) Manhattan plot generated by the MLM model; (**b**) Q-Q plot generated by the MLM model (λGC = 0.924); (**c**) Manhattan plot generated by the BLINK model; (**d**) Q-Q plot generated by the BLINK model (λGC = 1.025). The horizontal line indicates the Bonferroni-corrected genome-wide significance threshold, *p* = 9.53 × 10^−7^ (−log_10_*p* = 6.02). In panel (**c**), the labeled BLINK signals are located on SSC7 at 97.61 Mb and 111.59 Mb and on SSC8 at 136.72 Mb. Alternating colors distinguish chromosomes in the Manhattan plots; the horizontal line marks the genome-wide significance threshold, and the red dashed diagonal line in the Q-Q plots indicates the null expectation.

For NR (Table 2, Figure 2), 36 significant association records were obtained, corresponding to 35 distinct loci, among which *rs709317845* was detected by both models. The MLM model detected 33 significant SNPs, all located on SSC7 and mainly concentrated within the 91.10–98.15 Mb region, forming a continuous association peak around *VRTN* and its upstream and downstream regions. This region involved, or was adjacent to, genes including *EIF2S1*, *PIGH*, *EXD2*, *DPF3*, *PSEN1*, *PTGR2*, *COQ6*, *BBOF1*, *ALDH6A1*, *LIN52*, *ABCD4*, *VRTN*, *SYNDIG1L*, *NPC2*, *ISCA2*, *LTBP2*, *AREL1*, and *PGF*. The three significant SNPs detected by the BLINK model were distributed on SSC7 and SSC8: *rs709317845* was located at SSC7:97,614,602 bp, approximately 0.11 kb from *VRTN*, and represented the most significant association locus for NR (*p* = 2.14 × 10^−76^); *rs319413074* was located at SSC7:111,590,057 bp near *ENSSSCG00000061380*; and *rs345314475* was located at SSC8:136,719,720 bp near *ENSSSCG00000058817*.

Among the 35 SNP loci, 13 were recorded in Animal QTLdb, including *rs709317845*, *rs80888936*, *rs55619011*, *rs342113326*, *rs336541600*, *rs328449950*, *rs343248943*, *rs336800275*, *rs3471405130*, *rs80894106*, *rs338741094*, *rs325775517*, and *rs318853936*. Specifically, upstream of *VRTN*, *rs709317845* reached significance in both the MLM and BLINK models and corresponded to carcass length, thoracic vertebral number, rib number, and longissimus muscle depth traits [3,7,21,22]; *rs80888936* corresponded to teat-number traits [23,24]; and *rs55619011*, *rs342113326*, *rs336541600*, and *rs328449950*, located at SSC7:97.19–97.36 Mb, all corresponded to rib-number traits [3]. Within and downstream of *VRTN*, *rs336800275*, *rs3471405130*, *rs325775517*, and *rs318853936* corresponded to rib-number traits [3]; *rs80894106* and *rs338741094* corresponded to teat-number traits [25,26]; and *rs343248943* corresponded to loin muscle area [27].

All 35 SNP loci were recorded in the PigBiobank database, and in addition to loci recorded in Animal QTLdb, the remaining loci were also associated with many economically important traits: *rs341379902* was related to meat-quality and carcass traits; *rs320291489*, *rs340647044*, *rs330764436*, *rs331553584*, and *rs325733099* were related to reproductive traits; *rs319198707* was related to backfat thickness, reproductive traits, and blood indicators; *rs319413074* was related to uterine capacity and carcass length; *rs345314475* was related to blood indicators and free cholesterol; *rs325924112*, *rs1113113109*, *rs342816013*, *rs320308899*, *rs338689180*, *rs325054404*, *rs327294346*, *rs336722251*, *rs81231076*, *rs319768098*, *rs329716593*, and *rs330652997* were all related to traits such as backfat thickness, teat number, and carcass length; and *rs330539298* was related to loin-eye depth and teat number.

**Figure 3:**
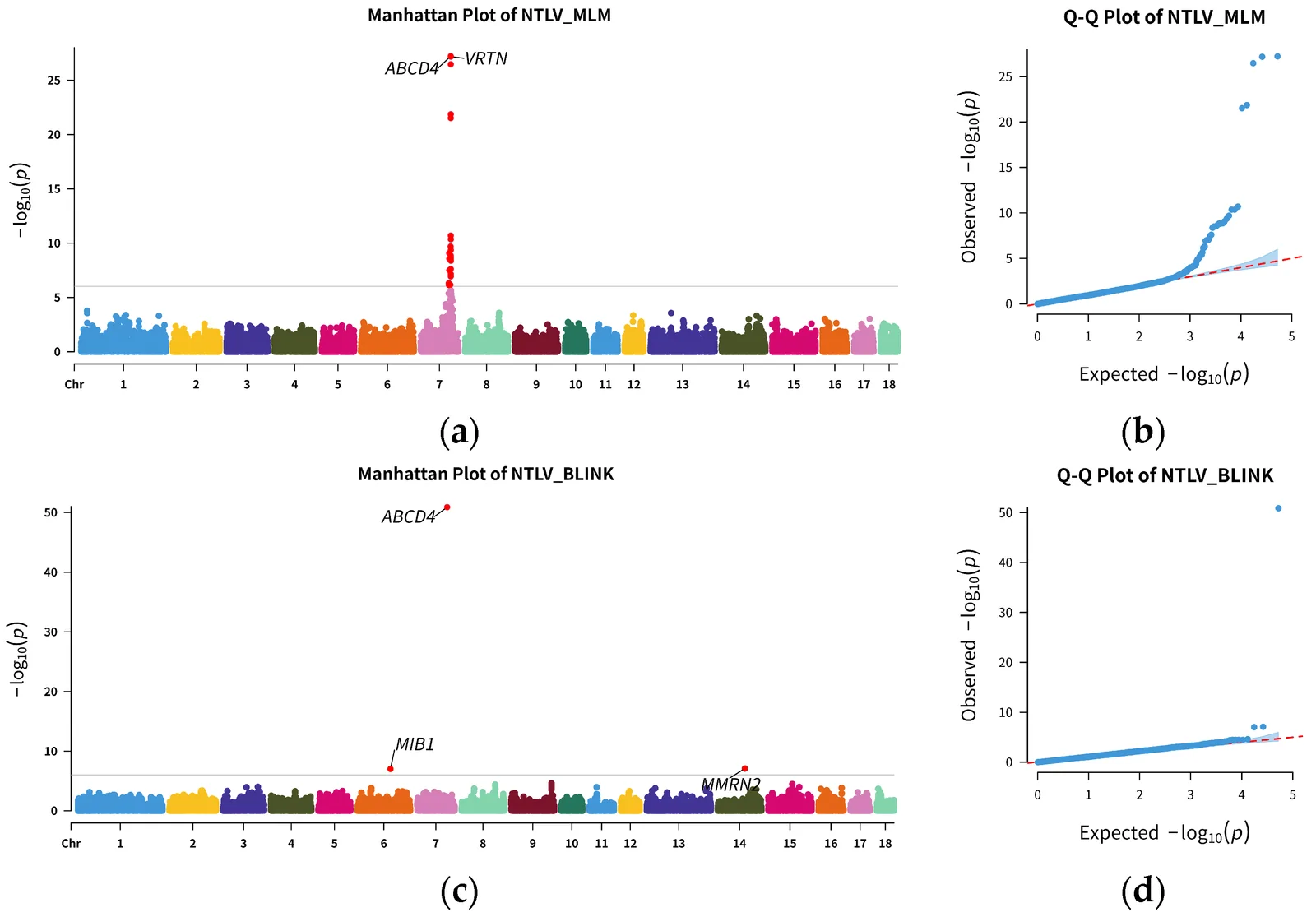
Manhattan and Q-Q plots for the total number of thoracolumbar vertebrae (NTLV). (**a**) Manhattan plot generated by the MLM model; (**b**) Q-Q plot generated by the MLM model (λGC = 0.989); (**c**) Manhattan plot generated by the BLINK model; (**d**) Q-Q plot generated by the BLINK model (λGC = 1.168). The horizontal line indicates the Bonferroni-corrected genome-wide significance threshold, *p* = 9.53 × 10^−7^ (−log_10_*p* = 6.02). Alternating colors distinguish chromosomes in the Manhattan plots; the horizontal line marks the genome-wide significance threshold, and the red dashed diagonal line in the Q-Q plots indicates the null expectation.

For NTLV (Table 3, Figure 3), 32 significant association records were listed, including 29 SNPs detected by the MLM model and three SNPs detected by the BLINK model. All significant loci detected by the MLM model were located within SSC7:91.19–98.15 Mb, covering or lying near genes including *PIGH*, *EXD2*, *PTGR2*, *ENTPD5*, *BBOF1*, *ALDH6A1*, *LIN52*, *ABCD4*, *VRTN*, *SYNDIG1L*, *NPC2*, *ISCA2*, *LTBP2*, *AREL1*, and *PGF*, and they highly overlapped with the SSC7 major peak identified for NR. The three loci detected by the BLINK model were located on SSC6, SSC7, and SSC14: *rs327309750* was located at SSC6:106,947,602 bp within *MIB1*; *rs1113113109* was located at SSC7:97,573,966 bp within *ABCD4* and represented the strongest BLINK signal for NTLV; and *rs3471622288* was located near *MMRN2*.

Animal QTLdb annotation showed that the NTLV-associated significant loci recorded in, or overlapping with records in, the database included *rs324614194*, *rs55619011*, *rs342113326*, *rs336541600*, *rs328449950*, *rs709317845*, *rs343248943*, *rs336800275*, *rs3471405130*, *rs80894106*, *rs338741094*, *rs325775517*, and *rs318853936.* Among these, *rs324614194* corresponded to loin muscle area and teat-number traits [23,24,27]; *rs55619011*, *rs342113326*, *rs336541600*, and *rs328449950*, located at SSC7:97.19–97.36 Mb, all corresponded to rib-number traits [3]; and *rs709317845* near *VRTN* corresponded to carcass length and straight carcass length [3,7,21,22]. In the *VRTN* and downstream segments, *rs343248943* corresponded to loin muscle area, teat number, and rib-number traits [3,13,23,27,28,29,30,31]; *rs336800275*, *rs3471405130*, *rs325775517*, and *rs318853936* corresponded to rib-number traits [25,32,33,34]; and *rs80894106* and *rs338741094* corresponded to teat-number traits [26,35]. These loci substantially overlapped with the QTLdb-supported loci for NR, indicating that the SSC7 major peak shared by NTLV and NR reflects not only differences in thoracic vertebrae/rib number but also overlap with trunk-development-related traits such as carcass length, muscle area, and teat number.

In the MLM results for NTLV, upstream SSC7 loci annotated in PigBiobank included *rs340647044*, *rs330764436*, and *rs331553584*, which were associated with reproductive traits; *rs341379902* and *rs325733099*, which were associated with loin-eye area; and *rs325924112* and *rs1113113109*, which were associated with traits such as backfat thickness and teat number. In the downstream SSC7:97.73–98.15 Mb segment, the annotated loci included *rs342816013* and *rs320308899*, both of which were associated with backfat thickness, loin-eye depth, teat number, and carcass length; *rs338689180*, *rs325054404*, *rs327294346*, *rs336722251*, and *rs81231076*, all of which were associated with backfat thickness, loin-eye depth, teat number, and carcass length; and *rs329716593* and *rs330652997*, which were associated with teat number and backfat thickness. The three BLINK-significant loci for NTLV were distributed on SSC6, SSC7, and SSC14: *rs327309750* was related to backfat thickness and teat number, *rs1113113109* was related to loin-eye depth, backfat thickness, and teat number, and *rs3471622288* was related to number born alive and loin-eye depth.

### 3.3. Candidate Gene Annotation

Based on annotation using the *Sus scrofa* 11.1 reference genome, Animal QTLdb (Release 59), and the PigBiobank database, the significant loci associated with NR and NTLV identified in this study can be classified into three major categories, and the core functional annotation focuses on several key candidate genes as follows.

The first category is the core SSC7:97.1–98.2 Mb region, with *VRTN* as the major-effect gene in this region. The significant loci in linkage with this region were highly associated with important economic traits in pigs, and QTLdb annotation indicated that this gene region mainly regulates carcass traits such as straight carcass length, loin muscle area, and rib number, making it a key candidate region affecting body skeletal structure and carcass characteristics.

The second category consists of several functional candidate genes flanking the SSC7 major peak, including *ABCD4*, *LTBP2*, *AREL1*, and *PGF*. A strongly significant association locus was located within *ABCD4*, and annotation indicated that this region was associated with loin-eye depth, backfat thickness, and teat-number traits. The *LTBP2* region was enriched with multiple consecutive significant SNPs, and functional annotation linked it to backfat thickness, loin-eye depth, teat number, carcass length, and related traits, suggesting that it is an important candidate gene for regulating carcass and teat development. In addition, several loci were annotated to the *AREL1* and *PGF* gene regions, although their functions require further validation.

The third category consists of independent candidate genes on SSC6, SSC8, and SSC14, with *MIB1* and *MMRN2* as the major candidates. A significant association locus was detected within *MIB1* on SSC6, and functional annotation linked it to backfat thickness and teat-number traits. The significant locus near *MMRN2* on SSC14 was associated with number born alive and loin-eye depth, suggesting potential regulatory roles in both reproductive and carcass traits.

Significant loci were also detected in regions containing *EXD2*, *PSEN1*, *EIF2S1*, *PIGH*, *NPC2*, and *ISCA2*. Among them, *EXD2* was associated with meat quality, meat-to-fat ratio, loin-eye area, and reproductive traits, whereas *PSEN1*, *NPC2*, and *ISCA2* mainly regulated carcass length, teat number, backfat thickness, loin-eye depth, and blood-related traits, making them auxiliary candidate genes for regulating carcass quality, meat quality, and physiological indicators in pigs.

## 4. Discussion

This study evaluated 389 Jishen Black pigs and found that the mean values of NR, NTLV, and NLV were 14.88, 20.94, and 6.05, respectively; NR was mainly concentrated at 15 ribs, NTLV at 21 vertebrae, and NLV at six lumbar vertebrae. This distribution indicates that rib number and total thoracolumbar vertebral number retain discrete variation suitable for genetic analysis in the Jishen Black pig population, whereas variation in lumbar vertebral number is relatively limited. Compared with the Landrace x Yorkshire crossbred population, in which NR and NTLV ranged from 14 to 16 ribs and from 19 to 23 vertebrae, respectively [4], the phenotypic range of Jishen Black pigs falls within the common range observed in commercial and synthetic pig populations; compared with reports in wild boars and Meishan pigs, which have approximately 19 thoracolumbar vertebrae, the mean NTLV of the present population is closer to that of synthetic populations that have undergone selection for body length and meat-production performance [36]. Moreover, previous studies have shown positive associations between NR and carcass length, straight carcass length, or meat value; therefore, the predominance of individuals with 15 ribs and 21 thoracolumbar vertebrae in Jishen Black pigs suggests that this population already has a certain long-carcass phenotypic basis while retaining an indigenous-pig genetic background.

In terms of genetic parameters, the heritability estimates for NR and NTLV in this study were 0.568 and 0.493, respectively, indicating moderate to high heritability, whereas NLV had a heritability of only 0.140. Studies have shown that the heritability estimates of NR and NTLV in a Landrace x Yorkshire crossbred population can reach 0.752 and 0.700 [4], and precise definition of rib number and vertebral number can also improve heritability estimates in commercial pig populations [37]. The heritability estimates in the present study were slightly lower than those reported in highly selected commercial populations, which may be related to the synthetic origin, sample size, population structure, and insufficient NLV variation in Jishen Black pigs. Nevertheless, the moderate to high heritability of NR and NTLV still indicates that these traits are mainly affected by additive genetic effects and are suitable for early selection through marker-assisted selection or genomic selection; in contrast, NLV showed limited individual variation and low additive variance in this population and is therefore not suitable as the main target for GWAS and marker development at the present stage. Similarly, the identification of loci and candidate genes associated with thoracic vertebral number in a Large White x Minzhu population also emphasizes the need to separately evaluate the genetic contributions of thoracic vertebrae/rib number and lumbar vertebral number in different genetic backgrounds [7].

Database annotation provided external evidence supporting the significant SNPs. Animal QTLdb curates published QTL, GWAS, eQTL, and related mapping information and can be used to determine whether significant SNPs fall within previously reported regions for rib number, carcass length, loin muscle area, or teat number [19], whereas PigBiobank integrates large-scale porcine phenotypic, genotypic, and omics data, thereby providing complementary annotation for loci associated with complex traits [20]. In this study, loci annotated in QTLdb involved traits such as rib number, carcass length, straight carcass length, loin muscle area, teat number, and left teat number, whereas loci annotated in PigBiobank were mainly related to backfat thickness, loin-eye depth, carcass length, teat number, number born alive, litter traits, blood indicators, free cholesterol, meat quality, meat-to-fat ratio, and population differentiation. These results suggest that the SSC7 major peak is not only associated with vertebral number but may also reflect a shared genetic background for body length, carcass composition, and teat number traits in this chromosomal region, whereas the SSC6-*MIB1* and SSC14-*MMRN2* loci may represent independent supplementary signals for NTLV. PigGTEx and large-scale meta-GWAS studies further indicate that interpretation of complex-trait loci in pigs should integrate tissue-specific expression, regulatory effects, and cross-population consistency [38].

SSC7:91–98 Mb was the core region shared by NR and NTLV in this study, in which *rs709317845* near *VRTN* showed the highest significance for NR, and *rs343248943*, *rs336800275*, and *rs3471405130* within *VRTN* repeatedly appeared for both traits. Previous studies have demonstrated in multiple populations that *VRTN* is associated with thoracic vertebral number, rib number, body length, and teat number, and that the *VRTN* insertion mutation exerts a strong effect on increasing thoracic vertebral/rib number [39]. In Suhuai pigs, *VRTN* g.20311_20312ins291 was significantly associated with rib number and carcass diagonal length [6]; functional studies in pigs and mice have shown that *VRTN* participates in thoracic vertebral and rib development, and *VRTN*-heterozygous mice can exhibit reduced numbers of thoracic vertebrae and ribs, possibly through Notch-related mechanisms affecting somitogenesis [40]. Therefore, the *VRTN* major peak detected in Jishen Black pigs in the present study is consistent with previous functional and population-association results, indicating that this region should be prioritized for subsequent marker validation and haplotype selection. However, genes such as *ABCD4*, *SYNDIG1L*, *LTBP2*, *AREL1*, and *PGF* are physically adjacent to *VRTN*, and the consecutive significant SNPs detected by the MLM model may partly reflect local linkage disequilibrium and should not be directly interpreted as multiple independent causal genes.

In addition to the SSC7 major peak, the *MIB1* signal on SSC6 also deserves attention. In this study, *rs327309750* was located within *MIB1* and reached genome-wide significance only for NTLV in the BLINK model, giving it relatively independent candidate value. A previous study in Beijing Black pigs linked *GREB1L* and *MIB1* on SSC6 to rib number and selected them, together with *ABCD4* on SSC7, as candidate genes [13]. Functionally, *MIB1* encodes the Mind bomb 1 E3 ubiquitin ligase, which promotes endocytosis of Delta/Jagged-like Notch ligands and thereby activates Notch signaling. Loss of *MIB1* in mice causes early embryonic lethality accompanied by multi-system defects involving somitogenesis, neurogenesis, angiogenesis, and cardiogenesis [41], and *MIB1* is indispensable for Notch signaling mediated by Jagged and Deltalike ligands [42]. Because somites are the embryonic basis for vertebral and rib development, the effect of *MIB1* on somitogenesis through Notch signaling provides clear developmental-biological support for its involvement in NTLV variation. Comparative evidence from humans and mice further indicates that Notch signaling is an important component of the mammalian segmentation clock and axial skeletal patterning [43]. Mouse studies have shown that loss of Notch activity disrupts somitogenesis, whereas variation in the level of Notch activity can alter the period of the segmentation clock [44,45]. In humans, *MIB1* is currently more commonly reported as a developmental disease gene related to the Notch pathway; for example, *MIB1* variants have been associated with left ventricular noncompaction cardiomyopathy and bicuspid aortic valve formation [46,47]. These findings indicate that the *MIB1*-Notch axis has a conserved role in the patterning of embryonic organs and tissues. Together with the SSC6-*MIB1* signal detected in this study, *MIB1* can be regarded as a priority candidate gene for NTLV in Jishen Black pigs.

*ABCD4* is located within the SSC7 major peak; *rs1113113109* represented the strongest BLINK signal for NTLV and was repeatedly significant in the MLM models for both NR and NTLV; Pairwise LD analysis using rs709317845 as the reference marker showed very strong LD with the *ABCD4* SNP rs1113113109 (r^2^ = 0.994) and heterogeneous LD with significant SNPs across *VRTN* (r^2^ = 0.434–0.931, excluding the reference marker), *SYNDIG1L* (r^2^ = 0.708), *NPC2* (r^2^ = 0.357), *ISCA2* (r^2^ = 0.427), *LTBP2* (r^2^ = 0.258–0.468), *AREL1* (r^2^ = 0.343–0.421), and *PGF* (r^2^ = 0.258) (Figure S2; Table S1). The LD heatmap therefore supports interpretation of the SSC7 peak as a linked regional signal rather than as evidence that every nearby gene represents an independent causal locus. The *LTBP2* region contained multiple consecutive significant SNPs for both NR and NTLV, and a study in Lijiang pigs linked polymorphisms in *VRTN*, *NR6A1*, and *LTBP2* to thoracic vertebral number [12]. Cross-species research has shown that Ltbp2-deficient mice exhibit multi-organ phenotypes involving body weight, fat mass, bone, and skin development, suggesting that *LTBP2* is associated with extracellular matrix and bone-tissue development [48]. By contrast, there is currently limited direct evidence connecting *MMRN2* on SSC14 with vertebral number in pigs; its PigBiobank annotation involved number born alive and loin-eye depth, and it is therefore better retained as an exploratory candidate signal for NTLV. Loci involving *EXD2*, *NPC2*, *ISCA2*, *AREL1*, and *PGF* overlapped with records for meat quality, backfat thickness, loin-eye depth, teat number, or rib number and thus have certain research value.

From the model results, the MLM model showed consecutive significant SNPs within the SSC7 major peak, indicating that NR and NTLV may be influenced by both major-effect loci and local linked haplotypes, whereas BLINK tended to detect a smaller number of strong signals, such as *VRTN*, *MIB1*, and *MMRN2*. From a breeding perspective, the moderate to high heritability of NR and NTLV and the stable detection of the SSC7 major-effect peak provide a basis for marker-assisted selection of carcass-length-related traits in Jishen Black pigs. Recent multi-population GWASs of carcass traits have shown that carcass composition and cutting traits are usually regulated by multiple QTLs [49]. Studies of commercial crossbred pigs have also shown that effective markers for cut weights and carcass-composition traits can be obtained through integrated GWAS and candidate-gene analyses [50]. Research on economically important carcass-cutting traits further emphasizes the application value of genome-wide markers in commercial pig populations [51]. Meta-GWAS results for loin muscle area indicate that muscle development and carcass-quality traits are also influenced by loci shared across populations [52]. Therefore, in practical breeding of Jishen Black pigs, the *VRTN* region should be evaluated together with rib number, total thoracolumbar vertebral number, carcass length, backfat thickness, loin-eye traits, and reproductive traits, to avoid selection based solely on a single vertebral-number trait.

Future studies could integrate single-cell or single-nucleus RNA sequencing of the presomitic mesoderm, newly formed somites, and developing vertebral tissues with chromatin-accessibility profiling, allele-specific expression, fine mapping, and functional perturbation. Such high-resolution transcriptomic and integrative analytical approaches may help characterize cell-type-specific regulatory networks and identify biologically relevant variants underlying the SSC7 association signal [53,54].

Several limitations should be acknowledged. The present study was based on 389 Jishen Black pigs from a single population, and the detected loci therefore primarily reflect the genetic background of this population. Although the major association signals were supported by comparisons between the MLM and BLINK models, LD analysis, and functional annotation, some model-specific associations should be interpreted with appropriate caution. Further evaluation in additional pig populations, together with haplotype, expression, and functional analyses, would help clarify the robustness and potential breeding relevance of these findings.

## 5. Conclusions

This study evaluated the genetic parameters of NR, NTLV, and NLV in 389 Jishen Black pigs and performed GWAS. NR and NTLV showed moderate to high heritability, whereas NLV had low heritability. GWAS identified a shared major-effect peak for NR and NTLV at SSC7:91.19–98.15 Mb, with *VRTN* and its adjacent linkage region serving as the core candidate region; *MIB1* on SSC6 and *MMRN2* on SSC14 were additional NTLV candidates, while *ABCD4*, *LTBP2*, *AREL1*, and *PGF* were retained as linked or exploratory candidates.

## Figures and Tables

**Table 1:** Descriptive statistics and variance-component estimates for NTLV, NR, and NLV in Jishen Black pigs (*n* = 389).

Trait	Mean ± SD	Min	Max	σ_a_^2^	σ_e_^2^	h^2^ ± SE
NTLV	20.94 ± 0.59	19	23	0.165	0.170	0.493 ± 0.104
NR	14.88 ± 0.63	14	16	0.224	0.170	0.568 ± 0.105
NLV	6.05 ± 0.39	5	8	0.022	0.135	0.140 ± 0.094

**Table 2:** Significant SNPs associated with NR.

SNP ID	Chr	Position (bp)	MAF	Effect (β)	SE	*p* Value	Nearest Gene	Distance (kb)	Model
*rs709317845*	7	97614602	0.311	0.725	—	2.14 × 10^−76^	*VRTN*	0.11	BLINK
*rs319413074*	7	111590057	0.063	0.296	—	2.95 × 10^−8^	*ENSSSCG00000061380*	0.14	BLINK
*rs345314475*	8	136719720	0.086	−0.237	—	7.36 × 10^−7^	*ENSSSCG00000058817*	0.74	BLINK
*rs320291489*	7	91098222	0.099	0.474	0.081	8.79 × 10^−9^	*EIF2S1*	Within	MLM
*rs340647044*	7	91195334	0.441	0.264	0.051	3.37 × 10^−7^	*ENSSSCG00000023791*	Within	MLM
*rs330764436*	7	91307815	0.150	0.430	0.067	5.27 × 10^−10^	*PIGH*	Within	MLM
*rs331553584*	7	92515376	0.278	0.410	0.056	1.03 × 10^−12^	*ENSSSCG00000049161*	0.67	MLM
*rs341379902*	7	92887462	0.289	0.368	0.053	1.35 × 10^−11^	*EXD2*	Within	MLM
*rs325733099*	7	92906034	0.294	0.373	0.051	2.03 × 10^−12^	*EXD2*	Within	MLM
*rs80888936*	7	96128654	0.353	0.278	0.052	1.66 × 10^−7^	*DPF3*	Within	MLM
*rs319198707*	7	96564735	0.294	−0.344	0.055	1.41 × 10^−9^	*PSEN1*	Within	MLM
*rs55619011*	7	97187301	0.437	0.388	0.049	3.84 × 10^−14^	*PTGR2*	Within	MLM
*rs342113326*	7	97271001	0.400	0.411	0.052	3.79 × 10^−14^	*COQ6*	Within	MLM
*rs336541600*	7	97319123	0.410	0.390	0.051	2.82 × 10^−13^	*BBOF1*	Within	MLM
*rs328449950*	7	97364568	0.410	0.390	0.051	2.82 × 10^−13^	*ALDH6A1*	Within	MLM
*rs325924112*	7	97490390	0.411	0.392	0.052	2.47 × 10^−13^	*LIN52*	Within	MLM
*rs1113113109*	7	97573966	0.312	0.716	0.052	4.43 × 10^−35^	*ABCD4*	Within	MLM
*rs709317845*	7	97614602	0.311	0.725	0.052	1.06 × 10^−35^	*VRTN*	0.11	MLM
*rs343248943*	7	97617907	0.427	0.544	0.050	2.11 × 10^−24^	*VRTN*	Within	MLM
*rs336800275*	7	97618186	0.328	0.680	0.051	5.28 × 10^−34^	*VRTN*	Within	MLM
*rs3471405130*	7	97623256	0.478	−0.403	0.048	5.87 × 10^−16^	*VRTN*	Within	MLM
*rs80894106*	7	97652632	0.392	0.564	0.049	1.76 × 10^−26^	*SYNDIG1L*	5.67	MLM
*rs342816013*	7	97730594	0.316	0.340	0.053	5.47 × 10^−10^	*NPC2*	Within	MLM
*rs320308899*	7	97741140	0.285	0.383	0.056	4.08 × 10^−11^	*ISCA2*	Within	MLM
*rs338689180*	7	97745450	0.279	0.430	0.057	2.82 × 10^−13^	*LTBP2*	Within	MLM
*rs325054404*	7	97745466	0.317	0.344	0.054	4.05 × 10^−10^	*LTBP2*	Within	MLM
*rs327294346*	7	97746336	0.317	0.344	0.054	4.05 × 10^−10^	*LTBP2*	Within	MLM
*rs336722251*	7	97746479	0.302	0.371	0.056	9.66 × 10^−11^	*LTBP2*	Within	MLM
*rs81231076*	7	97747046	0.317	0.344	0.054	4.05 × 10^−10^	*LTBP2*	Within	MLM
*rs338741094*	7	97752476	0.279	0.421	0.056	5.11 × 10^−13^	*LTBP2*	Within	MLM
*rs319768098*	7	97794678	0.310	0.273	0.054	8.57 × 10^−7^	*LTBP2*	Within	MLM
*rs325775517*	7	97892132	0.465	0.336	0.050	6.23 × 10^−11^	*AREL1*	Within	MLM
*rs329716593*	7	97909776	0.425	0.384	0.050	2.15 × 10^−13^	*AREL1*	Within	MLM
*rs330652997*	7	97909938	0.425	0.384	0.050	2.15 × 10^−13^	*AREL1*	Within	MLM
*rs318853936*	7	98151501	0.353	0.316	0.051	1.62 × 10^−9^	*PGF*	Within	MLM
*rs330539298*	7	104014060	0.103	−0.409	0.082	9.17 × 10^−7^	*ENSSSCG00000045310*	0.61	MLM

Note: SE, standard error; “—”, not available because GAPIT does not report standard errors for BLINK estimates.

**Table 3:** Significant SNPs associated with NTLV.

SNP ID	Chr	Position (bp)	MAF	Effect (β)	SE	*p* Value	Nearest Gene	Distance (kb)	Model
*rs327309750*	6	106947602	0.428	0.148	—	9.78 × 10^−8^	*MIB1*	Within	BLINK
*rs1113113109*	7	97573966	0.312	0.564	—	1.32 × 10^−51^	*ABCD4*	Within	BLINK
*rs3471622288*	14	87900906	0.474	−0.165	—	8.23 × 10^−8^	*MMRN2*	Within	BLINK
*rs340647044*	7	91195334	0.441	0.237	0.046	4.80 × 10^−7^	*ENSSSCG00000023791*	Within	MLM
*rs330764436*	7	91307815	0.150	0.311	0.062	7.23 × 10^−7^	*PIGH*	Within	MLM
*rs331553584*	7	92515376	0.278	0.321	0.051	8.35 × 10^−10^	*ENSSSCG00000049161*	0.67	MLM
*rs341379902*	7	92887462	0.289	0.274	0.048	3.09 × 10^−8^	*EXD2*	Within	MLM
*rs325733099*	7	92906034	0.294	0.287	0.047	2.77 × 10^−9^	*EXD2*	Within	MLM
*rs324614194*	7	96278617	0.148	0.305	0.060	6.98 × 10^−7^	*ENSSSCG00000043323*	Within	MLM
*rs55619011*	7	97187301	0.437	0.295	0.045	2.04 × 10^−10^	*PTGR2*	Within	MLM
*rs342113326*	7	97271001	0.400	0.289	0.048	3.10 × 10^−9^	*ENTPD5*	Within	MLM
*rs336541600*	7	97319123	0.410	0.292	0.047	1.44 × 10^−9^	*BBOF1*	Within	MLM
*rs328449950*	7	97364568	0.410	0.292	0.047	1.44 × 10^−9^	*ALDH6A1*	Within	MLM
*rs325924112*	7	97490390	0.411	0.293	0.047	1.40 × 10^−9^	*LIN52*	Within	MLM
*rs1113113109*	7	97573966	0.312	0.567	0.048	6.10 × 10^−28^	*ABCD4*	Within	MLM
*rs709317845*	7	97614602	0.311	0.567	0.048	6.71 × 10^−28^	*VRTN*	0.11	MLM
*rs343248943*	7	97617907	0.427	0.467	0.045	3.04 × 10^−22^	*VRTN*	Within	MLM
*rs336800275*	7	97618186	0.328	0.542	0.046	3.41 × 10^−27^	*VRTN*	Within	MLM
*rs3471405130*	7	97623256	0.478	−0.302	0.044	2.05 × 10^−11^	*VRTN*	Within	MLM
*rs80894106*	7	97652632	0.392	0.470	0.045	1.36 × 10^−22^	*SYNDIG1L*	5.68	MLM
*rs342816013*	7	97730594	0.316	0.266	0.049	1.17 × 10^−7^	*NPC2*	Within	MLM
*rs320308899*	7	97741140	0.285	0.295	0.052	2.42 × 10^−8^	*ISCA2*	Within	MLM
*rs338689180*	7	97745450	0.279	0.321	0.052	2.02 × 10^−9^	*LTBP2*	Within	MLM
*rs325054404*	7	97745466	0.317	0.268	0.049	9.84 × 10^−8^	*LTBP2*	Within	MLM
*rs327294346*	7	97746336	0.317	0.268	0.049	9.84 × 10^−8^	*LTBP2*	Within	MLM
*rs336722251*	7	97746479	0.302	0.282	0.051	7.01 × 10^−8^	*LTBP2*	Within	MLM
*rs81231076*	7	97747046	0.317	0.268	0.049	9.84 × 10^−8^	*LTBP2*	Within	MLM
*rs338741094*	7	97752476	0.279	0.314	0.052	3.03 × 10^−9^	*LTBP2*	Within	MLM
*rs325775517*	7	97892132	0.465	0.290	0.045	4.36 × 10^−10^	*AREL1*	Within	MLM
*rs329716593*	7	97909776	0.425	0.310	0.046	4.40 × 10^−11^	*AREL1*	Within	MLM
*rs330652997*	7	97909938	0.425	0.310	0.046	4.40 × 10^−11^	*AREL1*	Within	MLM
*rs318853936*	7	98151501	0.353	0.281	0.047	4.20 × 10^−9^	*PGF*	Within	MLM

Note: SE, standard error; “—”, not available because GAPIT does not report standard errors for BLINK estimates.

## Data Availability

The data presented in this study are available on request from the corresponding authors due to the need to ensure standardized and traceable data use, as well as to prevent inappropriate use or unauthorized citation.

## Supplementary Materials

The following supporting information can be downloaded at: https://www.mdpi.com/article/10.3390/vetsci13080776/s1, Table S1: Pairwise r^2^ values between the NR lead SNP rs709317845 and prioritized significant SNPs within the SSC7 core interval; Figure S1: Principal component analysis and scree plot of the Jishen Black pig population, including (a) a three-dimensional PCA plot based on PC1, PC2, and PC3 and (b) a scree plot showing the variance explained by the first 10 principal components; Figure S2: Linkage-disequilibrium structure across the SSC7:97.57–98.15 Mb core association interval, showing pairwise D′ values among significant SNPs surrounding *VRTN* and adjacent genes.

## Abbreviations

BLINK	Bayesian-information and Linkage-disequilibrium Iteratively Nested Keyway
GWAS	Genome-wide association study
MAF	Minor allele frequency
MLM	Mixed linear model
NLV	Number of lumbar vertebrae
NR	Number of ribs
NTLV	Total number of thoracolumbar vertebrae
PCA	Principal component analysis
QTL	Quantitative trait locus
SNP	Single-nucleotide polymorphism
SSC	*Sus scrofa* chromosome
