# Supplementary material for: Genome-Wide Association Study of Rib Number and Total Thoracolumbar Vertebrae Number in a Jishen Black Pig Population
Source: Vet Sci. 2026 Aug 3;13(8):776. doi: 10.3390/vetsci13080776 (PMC13517530; doi:10.3390/vetsci13080776)
Supplement: Supplementary file 1 [file vetsci-13-00776-s001.zip › vetsci-4443800-Table S1.pdf]

Table S1. Pairwise r^2 values between the NR lead SNP *rs709317845* and prioritized significant SNPs in the SSC7 core interval.

**SNP ID**	**Position**	**Gene**	**LD with rs709317845(r^2)**
rs1113113109	97,573,966	*ABCD4*	0.994
rs343248943	97,617,907	*VRTN*	0.634
rs336800275	97,618,186	*VRTN*	0.931
rs3471405130	97,623,256	*VRTN*	0.434
rs80894106	97,652,632	*SYNDIG1L*	0.708
rs342816013	97,730,594	*NPC2*	0.357
rs320308899	97,741,140	*ISCA2*	0.427
rs338689180	97,745,450	*LTBP2*	0.468
rs325054404	97,745,466	*LTBP2*	0.354
rs327294346	97,746,336	*LTBP2*	0.354
rs336722251	97,746,479	*LTBP2*	0.397
rs81231076	97,747,046	*LTBP2*	0.354
rs338741094	97,752,476	*LTBP2*	0.463
rs319768098	97,794,678	*LTBP2*	0.258
rs325775517	97,892,132	*AREL1*	0.343
rs329716593	97,909,776	*AREL1*	0.421
rs330652997	97,909,938	*AREL1*	0.421
rs318853936	98,151,501	*PGF*	0.258
